# Supplementary material for: A Salmonella Typhi RNA thermosensor regulates virulence factors and innate immune evasion in response to host temperature
Source: PLoS Pathog. 2021 Mar 2;17(3):e1009345. doi: 10.1371/journal.ppat.1009345 (PMC7954313; doi:10.1371/journal.ppat.1009345)
Supplement: S1 Table — Hard constraints for RNA structure prediction. (DOCX) [file ppat.1009345.s002.docx]

**Table S1: Related to Figure 3. Hard constraints for RNA structure prediction**

| **Structural property** | **Mfold constraints** [1] |
| --- | --- |
| GGG26-28 must be unpaired | P 26 0 3 |
| UG52-53 must be unpaired | P 52 0 2 |
| AG73-74 must be unpaired | P 73 0 2 |
| GG97-98 must be unpaired | P 97 0 3 |
| GAAG106-109 must be unpaired | P 106 0 4 |
| G135 must be unpaired | P 135 0 1 |
| A31 must be paired | F 31 0 1 |
| A39 must be paired | F 39 0 1 |
| UG41-42 must be paired | F 41 0 2 |
| G46 must be paired | F 46 0 1 |
| AA49-50 must be paired | F 49 0 2 |
| G80 must be paired | F 80 0 1 |
| AG117-118 must be paired | F 101 0 1 |
| GGAG124-127 must be paired | F 124 0 4 |

**Reference**

1. Zuker M. Mfold web server for nucleic acid folding and hybridization prediction. Nucleic Acids Res. 2003 Jul 1;31(13):3406–15.
