# Supplementary material for: A Salmonella Typhi RNA thermosensor regulates virulence factors and innate immune evasion in response to host temperature
Source: PLoS Pathog. 2021 Mar 2;17(3):e1009345. doi: 10.1371/journal.ppat.1009345 (PMC7954313; doi:10.1371/journal.ppat.1009345)
Supplement: S2 Table — (DOCX) [file ppat.1009345.s003.docx]

**Table S2: Bacterial strains used in this study**

| Bacterial strain | Description | Origin |
| --- | --- | --- |
| *Salmonella* *enterica* serovar Typhi |  |  |
| Ty2 | Wild-type | Monack lab strain collection |
| SMB4 | Ty2(pSLTS) | This study |
| SMB9 | Ty2 *tviA* 5’ UTR T90,92C mutations; *tviA*-REP | This study |
| SMB24 | Ty2 Δ*fliC* | This study |
| SMB28 | SMB9 Δ*fliC* | This study |
| SMB39 | Ty2 Δ*tviA* | This study |
| SMB73 | Ty2 *tviA* 5’ UTR T89,91G;C93G mutations; *tviA*-DEREP | This study |
|  |  |  |
| *Escherichia coli* |  |  |
| DH5⍺ | *supE44*, Δ*lacU169* (ψ80*lacZ*Δ*M15*), *hsdR17*, *recA1*, *gyrA96*, *thi1*, *relA1* | Monack lab strain collection |
