# Supplementary material for: A Salmonella Typhi RNA thermosensor regulates virulence factors and innate immune evasion in response to host temperature
Source: PLoS Pathog. 2021 Mar 2;17(3):e1009345. doi: 10.1371/journal.ppat.1009345 (PMC7954313; doi:10.1371/journal.ppat.1009345)
Supplement: S3 Table — (DOCX) [file ppat.1009345.s004.docx]

**Table S3: Oligonucleotides used in this study^a^**

| Primer/gBlock name | Sequence (5’ to 3’) | Description |
| --- | --- | --- |
| tviA_NheI_fw | AAAATGCTAGCTAGGCCGTTAGTACTATTAAAATTAG | forward primer to amplify the *tviA* (*t4353*) 5’-UTR plus coding region (-82 to +3 bp from *tviA* ATG) to make plasmid pBO4421 |
| tviA_EcoRI_rv | AAGAATTCCATGAAGTCTCCTTATGCTGAAAT | reverse primer to amplify the *tviA* (*t4353*) 5’-UTR plus coding region (-82 to +3 bp from *tviA* ATG) to make plasmid pBO4421 |
| tviA_rep1_fw | gatattgcttgtaccTCttctatggatgaaattag | mutagenesis forward primer to introduce the mutation T90C (rep1) into *tviA* (*t4353*) 5’-UTR to make plasmid pBO4427 |
| tviA_rep1_rev | CTAATTTCATCCATAGAAGAGGTACAAGCAATATC | mutagenesis reverse primer to introduce the mutation T90C (rep1) into *tviA* (*t4353*) 5’-UTR to make plasmid pBO4427 |
| tviA_rep2_fw | gatattgcttgtaccTttCctatggatgaaattag | mutagenesis forward primer to introduce the mutation T92C (rep2) into *tviA* (*t4353*) 5’-UTR to make plasmid pBO4428 |
| tviA_rep2_rev | CTAATTTCATCCATAGGAAAGGTACAAGCAATATC | mutagenesis reverse primer to introduce the mutation T92C (rep2) into *tviA* (*t4353*) 5’-UTR to make plasmid pBO4428 |
| tviA_rep3_fw | gatattgcttgtaccTCtCctatggatgaaattag | mutagenesis forward primer to introduce the mutation T90,92C (rep3) into *tviA* (*t4353*) 5’-UTR to make plasmids pBO4424, pBO4447 |
| tviA_rep3_rev | CTAATTTCATCCATAGGAGAGGTACAAGCAATATC | mutagenesis reverse primer to introduce the mutation T90,92C (rep3) into *tviA* (*t4353*) 5’-UTR to make plasmids pBO4424, pBO4447 |
| tviA_derep_fw | gatattgcttgtaccGTGtGtatggatgaaattag | mutagenesis forward primer to introduce the mutation T89,91G,C93G (derep) into *tviA* (*t4353*) 5’-UTR to make plasmids pBO4426, pBO4448 |
| tviA_derep_rev | CTAATTTCATCCATACACACGGTACAAGCAATATC | mutagenesis reverse primer to introduce the mutation T89,91G,C93G (derep) into *tviA* (*t4353*) 5’-UTR to make plasmids pBO4426, pBO4448 |
| tviA_RO_T7_fw | TGAAATTAATACGACTCACTATAGGTAGGCCGTTAGTACTATTAAAATTA | forward primer to amplify the *tviA* (*t4353*) 5’-UTR plus coding region (-82 to +60 bp from *tviA* ATG); run-off plasmid; T7 promoter to make plasmid pBO4439 |
| tviA_RO_NaeI_rv | AGCCGGCGCCAGCAGCTCCAACC | reverse primer to amplify the *tviA* (*t4353*) 5’-UTR plus coding region (-82 to +60 bp from *tviA* ATG); run-off plasmid; NaeI restriction site to make plasmid pBO4439 |
| MF | ATCTCAAGAGTGGCAGC | Forward primer to amplify selection cassette from pT2SC for scarless mutagenesis mutation cassette plasmid construction;[1] |
| MR | TTACGCCCCGCCCTGC | Reverse primer to amplify selection cassette from pT2SC for scarless mutagenesis mutation cassette plasmid construction; [1] |
| pHAfor | CGCAGGAAAGAACATGTG | Forward primer to amplify backbone from pUC19 for scarless mutagenesis mutation cassette plasmid construction; [1] |
| pHArev | AAGGGCCTCGTGATACG | Reverse primer to amplify backbone from pUC19 for scarless mutagenesis mutation cassette plasmid construction; [1] |
| pHA.seq.F | TATCAGGGTTATTGTCTCATGAGCG | Sequencing primer to verify mutation cassette plasmid; [1] |
| pHA.seq.R | ACTTGAGCGTCGATTTTTGTGATGC | Sequencing primer to verify mutation cassette plasmid; [1] |
| tviA_for_qRT | TCGACTATGTATCGCTGGAGA | Forward qRT-PCR primer to assess *tviA* transcript levels; [2] |
| tviA_rev_qRT | GAATCCGGCAATAACAGATAG | Reverse qRT-PCR primer to assess *tviA* transcript levels; [2] |
| bgaB_for_2 | CGGCGACTGCAACTACTC | Forward qRT-PCR primer to assess *bgaB* transcript levels |
| bgaB_rev_2 | CGCGACAGCACAATTCTCA | Reverse qRT-PCR primer to assess *bgaB* transcript levels |
| U16SRT-F | ACTCCTACGGGAGGCAGCAGT | Forward universal 16S qRT-PCR primer; [3] |
| U16SRT-R | TATTACCGCGGCTGCTGGC | Reverse universal 16S qRT-PCR primer; [3] |
| tviA-F | TAGGCCGTTAGTACTATTAA | Forward PCR primer to amplify mutation cassette from pSMB11 and pSMB12; also used for Sanger sequencing verification of introduction of point mutations into Ty2 |
| tviA-R | CAACCCCGAAATAGATATCA | Reverse PCR primer to amplify mutation cassette from pSMB11 and pSMB12; also used for Sanger sequencing verification of introduction of point mutations into Ty2 |
| *tviA*-REP 5’ mutation fragment | AGGCGTATCACGAGGCCCTTTAGGCCGTTAGTACTATTAAAATTAGGGTAATAATTTTATTGTTAGTTAATTGTTAACAGGAGCAAAGAATTAGATATTGCTTGTACCTCTCCTATGGATGAAATATCTCAAGAGTGGCAGCGGT | gBlock sequence used to generate pSMB11 |
| *tviA*-REP 3’ mutation fragment | GCAGGGCGGGGCGTAAATATTGCTTGTACCTCTCCTATGGATGAAATTAGGTTATTTCAGCATAAGGAGACTTCATGAGGTTTCATCATTTCTGGCCTCCGAATGATATCTATTTCGGGGTTGCGCAGGAAAGAACATGTGAG | gBlock sequence used to generate pSMB11 |
| oSMB64 | TGTTTTCGATGCGGCAACATCA | Forward PCR primer to check for presence of Ty2 *viaB* genes; use with oSMB65 |
| oSMB65 | TAAAGCCTGCTACGCCAGTGAT | Reverse PCR primer to check for presence of Ty2 *viaB* genes; use with oSMB64 |
| oSMB67 | CGGCAATCTGGAGGCAAAGTTT | PCR primer to verify Ty2 Δ*fliC*; use with oSMB68 |
| oSMB68 | GGCAAGACTCAGGGAGTTACGT | PCR primer to verify Ty2 Δ*fliC*; use with oSMB67 |
| oSMB69 | TTCATTTCCGAAGCAGTCACGC | PCR primer to amplify around Ty2 *tviA* 5’ UTR for Sanger sequencing verification of introduction of point mutations; use with oSMB70 |
| oSMB70 | GCGGCTCACGTACTCCAAATTT | PCR primer to amplify around Ty2 *tviA* 5’ UTR for Sanger sequencing verification of introduction of point mutations; use with oSMB69 |
| oSMB75 | GGTTTCCGCTGGTCTACAAAGC | PCR primer to check for presence of Ty2 *viaB* genes; use with oSMB76 |
| oSMB76 | ACCGAGCTCTTTTTCCATCCGA | PCR primer to check for presence of Ty2 *viaB* genes; use with oSMB75 |
| oSMB82 | AGGCGTATCACGAGGCCCTTTGCGCGGAATAATGATGCATAAAGC | PCR primer to amplify part of Ty2 gDNA sequence to generate mutation cassette plasmid pSMB5; used with oSMB83 |
| oSMB83 | ACCGCTGCCACTCTTGAGATGAATCAATCGCCGGAGATCTTTTCCTTATCAATTACAACTTGATGTTATTGGG | PCR primer to amplify part of Ty2 gDNA sequence to generate mutation cassette plasmid pSMB5; used with oSMB82 |
| oSMB84 | GCAGGGCGGGGCGTAAGATAAGGAAAAGATCTCCGGCGATTGATTCACCGAC | PCR primer to amplify part of Ty2 gDNA sequence to generate mutation cassette plasmid pSMB5; used with oSMB85 |
| oSMB85 | CTCACATGTTCTTTCCTGCGCCGTCGTCTTATCCAGCGTGA | PCR primer to amplify part of Ty2 gDNA sequence to generate mutation cassette plasmid pSMB5; used with oSMB84 |
| oSMB86 | AGGCGTATCACGAGGCCCTTTTGTGTAAAAATCCCGTTTAGGCC | PCR primer to amplify part of Ty2 gDNA sequence to generate mutation cassette plasmid pSMB9; used with oSMB105 |
| oSMB95 | TGCGCGGAATAATGATGCATAAAGC | PCR primer to amplify mutation cassette from pSMB5 |
| oSMB96 | CCGTCGTCTTATCCAGCGTGA | PCR primer to amplify mutation cassette from pSMB5 |
| oSMB97 | TTGTGTAAAAATCCCGTTTAGGCC | PCR primer to amplify mutation cassette from pSMB9 |
| oSMB105 | ACCGCTGCCACTCTTGAGATATAAAAATTACCGCAGAAGTCTCCTTATGCTGAAATAACCTAATTTC | PCR primer to amplify part of Ty2 gDNA sequence to generate mutation cassette plasmid pSMB9; used with oSMB86 |
| oSMB106 | GCAGGGCGGGGCGTAAGCATAAGGAGACTTCTGCGGTAATTTTTATTGAGTAAAACACG | PCR primer to amplify part of Ty2 gDNA sequence to generate mutation cassette plasmid pSMB9; used with oSMB107 |
| oSMB107 | CTCACATGTTCTTTCCTGCGCTAGGTGTTTATTCAGATACGCTACC | PCR primer to amplify part of Ty2 gDNA sequence to generate mutation cassette plasmid pSMB9; used with oSMB106 |
| oSMB111 | CTAGGTGTTTATTCAGATACGCTACC | PCR primer to amplify mutation cassette from pSMB9 |
| oSMB113 | GGCGGGGCGTAAATATTGCTTGTACCGTGTGTATGGATGAAATTAGG | QuikChange Lightning primer to generate plasmid pSMB12 |
| oSMB114 | GATATTGCTTGTACCGTGTGTATGGATGAAATATCTCAAGAGTGGCAGC | QuikChange Lightning primer to generate plasmid pSMB12 |
| oSMB117 | ATCCCGTTTAGGCCGTTAGT | PCR primer to verify Ty2 Δ*tviA* |
| oSMB118 | TCAGATACGCTACCGCCC | PCR primer to verify Ty2 Δ*tviA* |

^a^Unless otherwise indicated, oligonucleotides were designed as part of this study

**References**

1. Kim J, Webb AM, Kershner JP, Blaskowski S, Copley SD. A versatile and highly efficient method for scarless genome editing in *Escherichia coli* and *Salmonella enterica*. BMC Biotechnol. 2014;14(1):84–13.

2. Winter SE, Winter MG, Thiennimitr P, Gerriets VA, Nuccio S-P, Rüssmann H, et al. The TviA auxiliary protein renders the *Salmonella enterica* serotype Typhi RcsB regulon responsive to changes in osmolarity. Mol Microbiol. 2009 Oct;74(1):175–93.

3. Clifford RJ, Milillo M, Prestwood J, Quintero R, Zurawski DV, Kwak YI, et al. Detection of Bacterial 16S rRNA and Identification of Four Clinically Important Bacteria by Real-Time PCR. PLoS ONE. 2012 Nov 6;7(11):e48558–6.
