# Supplementary material for: A Salmonella Typhi RNA thermosensor regulates virulence factors and innate immune evasion in response to host temperature
Source: PLoS Pathog. 2021 Mar 2;17(3):e1009345. doi: 10.1371/journal.ppat.1009345 (PMC7954313; doi:10.1371/journal.ppat.1009345)
Supplement: S4 Table — (DOCX) [file ppat.1009345.s005.docx]

**Table S4. Plasmids used in this study**

| Plasmid name | Description | Reference/Origin |
| --- | --- | --- |
| pUC18 | Amp^R^; cloning vector | [1] |
| pUC19 | Amp^R^; template for amplification of origin of replication and ampicillin resistance gene for scarless mutagenesis | [1]; Addgene_50005 |
| pBAD2-*bgaB*-His | *bgaB* reporter gene vector, Ap^r^, *araC*, P*_BAD_* promoter, His‑Tag at the C‑terminal of BgaB | [2] |
| pBO3146 | pBAD2-*bgaB*-His; ICR between pYV0075(*yscW*) and pYV0076(*lcrF*) plus 9 bp of *lcrF* coding region (123 to +9 bp from *lcrF* ATG) | [2] |
| pBO4421 | pBAD2-*bgaB*-His; 5’-UTR of *tviA* (*t4353*) 5’-UTR plus coding region (-103 to +3 bp from *tviA* ATG) | This study |
| pBO4424 | pBAD2-*bgaB*-His; 5’-UTR of *tviA* (*t4353*) 5’-UTR plus coding region (-103 to +3 bp from *tviA* ATG); mutant rep3 (T90,92C) | This study |
| pBO4426 | pBAD2-*bgaB*-His; 5’-UTR of *tviA* (*t4353*) 5’-UTR plus coding region (-103 to +3 bp from *tviA* ATG); mutant derep4 (T89,91G,C93G) | This study |
| pBO4427 | pBAD2-*bgaB*-His; 5’-UTR of *tviA* (*t4353*) 5’-UTR plus coding region (-103 to +3 bp from *tviA* ATG); mutant rep1 (T90C) | This study |
| pBO4428 | pBAD2-*bgaB*-His; 5’-UTR of *tviA* (*t4353*) 5’-UTR plus coding region (-103 to +3 bp from *tviA* ATG); mutant rep2 (T92C) | This study |
| pBO4439 | pUC18; 5’-UTR of *tviA* (*t4353*) 5’-UTR plus coding region (-103 to +60 bp from *tviA* ATG); run-off plasmid for structure probing and primer extension inhibition | This study |
| pBO4447 | pUC18; 5’-UTR of *tviA* (*t4353*) 5’-UTR plus coding region (-103 to +60 bp from *tviA* ATG); run-off plasmid for structure probing and primer extension inhibition; mutant rep3 (T90,92C) | This study |
| pBO4448 | pUC18; 5’-UTR of *tviA* (*t4353*) 5’-UTR plus coding region (-103 to +60 bp from *tviA* ATG); run-off plasmid for structure probing and primer extension inhibition; mutant derep (T89,91G,C93G) | This study |
| pSLTS | Ori SC101(Ts) Amp^R^; P_araB_ for λ-Red; P_tetR_ for I-SceI | [3]; Addgene_59386 |
| pT2SC | Amp^R^ Cm^R^; template for amplification of selection cassette that contains chloramphenicol resistance gene and I-SceI cut site | [3]; Addgene_59382 |
| pSMB5 | Amp^R^ Cm^R^; mutation cassette plasmid for knocking out *fliC* in *S.* Typhi using scarless mutagenesis | This study |
| pSMB9 | Amp^R^ Cm^R^; mutation cassette plasmid for knocking out *tviA* in *S.* Typhi using scarless mutagenesis | This study |
| pSMB11 | Amp^R^ Cm^R^; mutation cassette plasmid for introducing repressing T90,92C point mutations into *tviA* 5’ UTR in *S.* Typhi using scarless mutagenesis | This study |
| pSMB12 | Amp^R^ Cm^R^; mutation cassette plasmid for introducing derepressing T89,91G;C93G point mutations into *tviA* 5’ UTR in *S.* Typhi using scarless mutagenesis | This study |

**References**

1. Yanisch-Perron C, Vieira J, Messing J. Improved M13 phage cloning vectors and host strains: nucleotide sequences of the M13mp18 and pUC19 vectors. Gene. 1985;33(1):103–19.

2. Righetti F, Nuss AM, Twittenhoff C, Beele S, Urban K, Will S, et al. Temperature-responsive *in vitro* RNA structurome of *Yersinia pseudotuberculosis*. Proc Natl Acad Sci USA. 2016 Jun 28;113(26):7237–42.

3. Kim J, Webb AM, Kershner JP, Blaskowski S, Copley SD. A versatile and highly efficient method for scarless genome editing in *Escherichia coli* and *Salmonella enterica*. BMC Biotechnol. 2014;14(1):84–13.
