# Supplementary figures and images for: A Salmonella Typhi RNA thermosensor regulates virulence factors and innate immune evasion in response to host temperature
Source: PLoS Pathog. 2021 Mar 2;17(3):e1009345. doi: 10.1371/journal.ppat.1009345 (PMC7954313; doi:10.1371/journal.ppat.1009345)

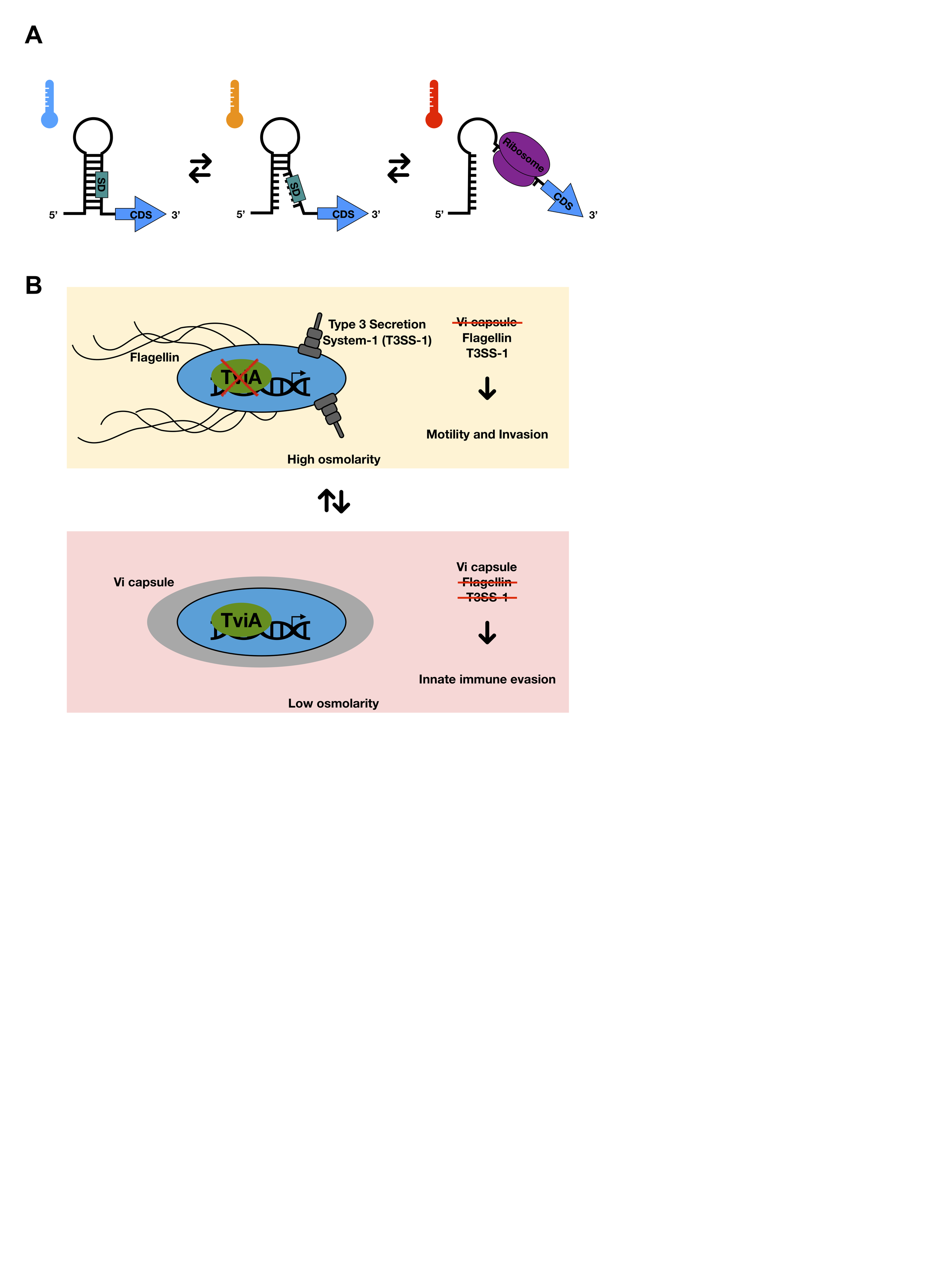

Supplement: S1 Fig — A) Schematic depicting RNAT function in response to temperature. At low temperatures, Watson-Crick base-pairing in the 5’ UTR of the mRNA generates secondary structure that prevents ribosome access to the SD region and translation. As the temperature increases, the nucleotide bonds melt until the structure is fully open, ribosomes can bind, and translation can occur. B) Schematic depicting effect of osmolarity on TviA production and subsequent virulence factor expression. Under high osmolarity conditions (e.g., intestinal lumen), transcription of tviA is repressed, meaning that S. Typhi has high expression of flagellin and type 3 secretion system-1 (T3SS-1) but does not express Vi capsule. This expression pattern renders S. Typhi motile and invasive. Transition to low osmolarity conditions (e.g., intestinal epithelium or intracellular) induces tviA transcription. TviA then induces Vi capsule expression and suppression of flagellin and T3SS-1 expression, which gives S. Typhi an immune evasive phenotype. (TIF) [file ppat.1009345.s006.tif]

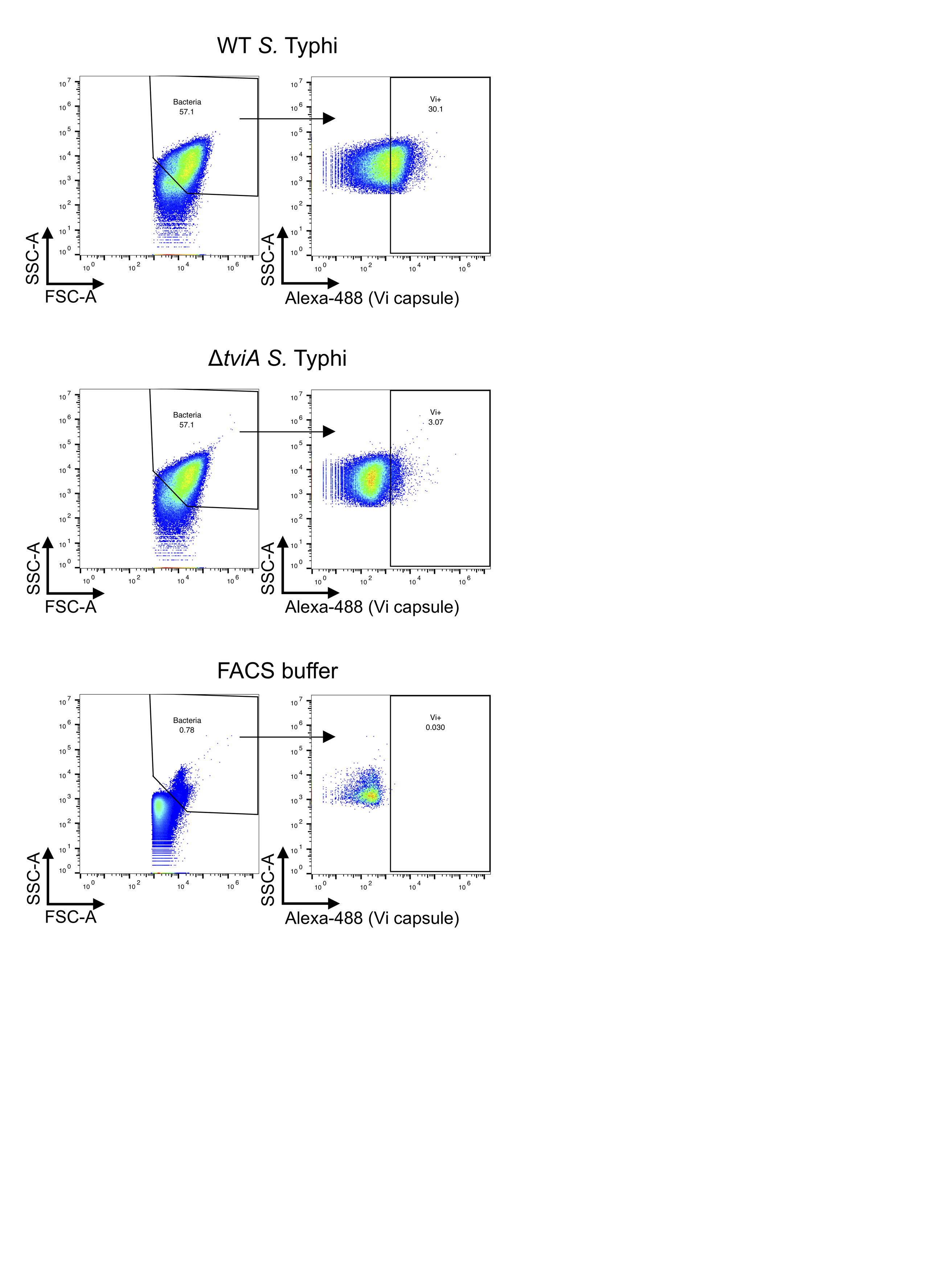

Supplement: S2 Fig — Example of gating strategy used to identify S. Typhi bacteria and quantitate level of Vi capsule expression following temperature shifts. (TIF) [file ppat.1009345.s007.tif]

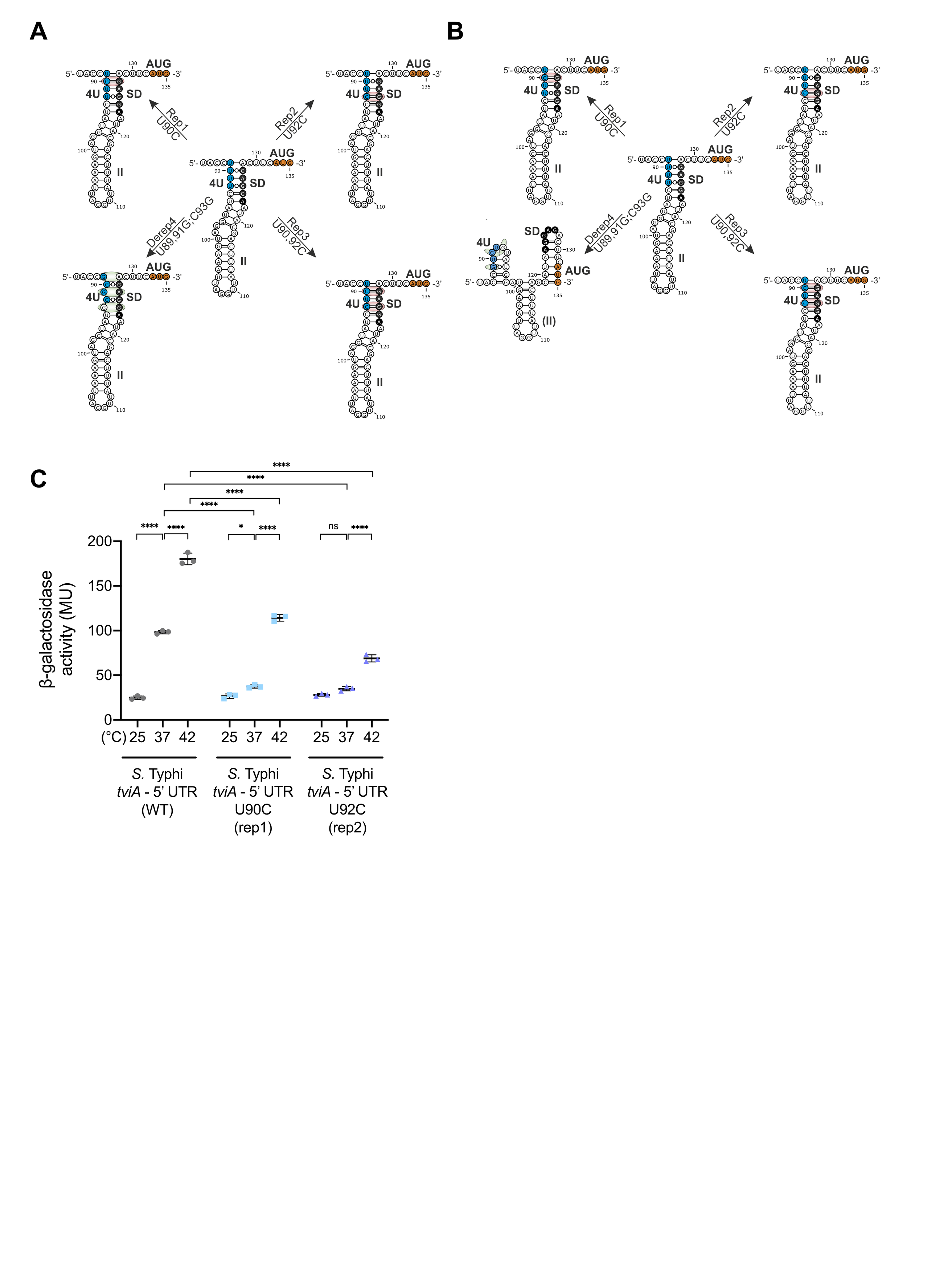

Supplement: S3 Fig — A and B) Schematic representation of the secondary structure of the tviA RNAT stem (nucleotides 85 to 132 plus AUG codon) including the fourU motif (4U; blue nucleotides), the SD region (black nucleotides), and the AUG codon (orange nucleotides). The desired (A) as well as the Mfold-predicted minimum free energy (B) repressed (mutations highlighted in red) and derepressed (mutations highlighted in green) RNAT structures resulting from nucleotide exchanges are depicted as well. C) Effect of altering base-pairing of the fourU region on temperature-dependent translation. Comparison of translation efficiency at different temperatures using the S. Typhi wildtype tviA 5’ UTR (WT), the rep1 (T90C) tviA 5’ UTR mutant, and the rep2 (T92C) tviA 5’ UTR mutant bgaB fusion constructs. Data shown are representative of 4 independent experiments (C) with triplicate samples for each condition. Data are represented as mean ± SD. NS, not significant. Statistical significance determined using two-way ANOVA with Tukey’s correction. * p < 0.05, **** p < 0.0001. (TIF) [file ppat.1009345.s008.tif]

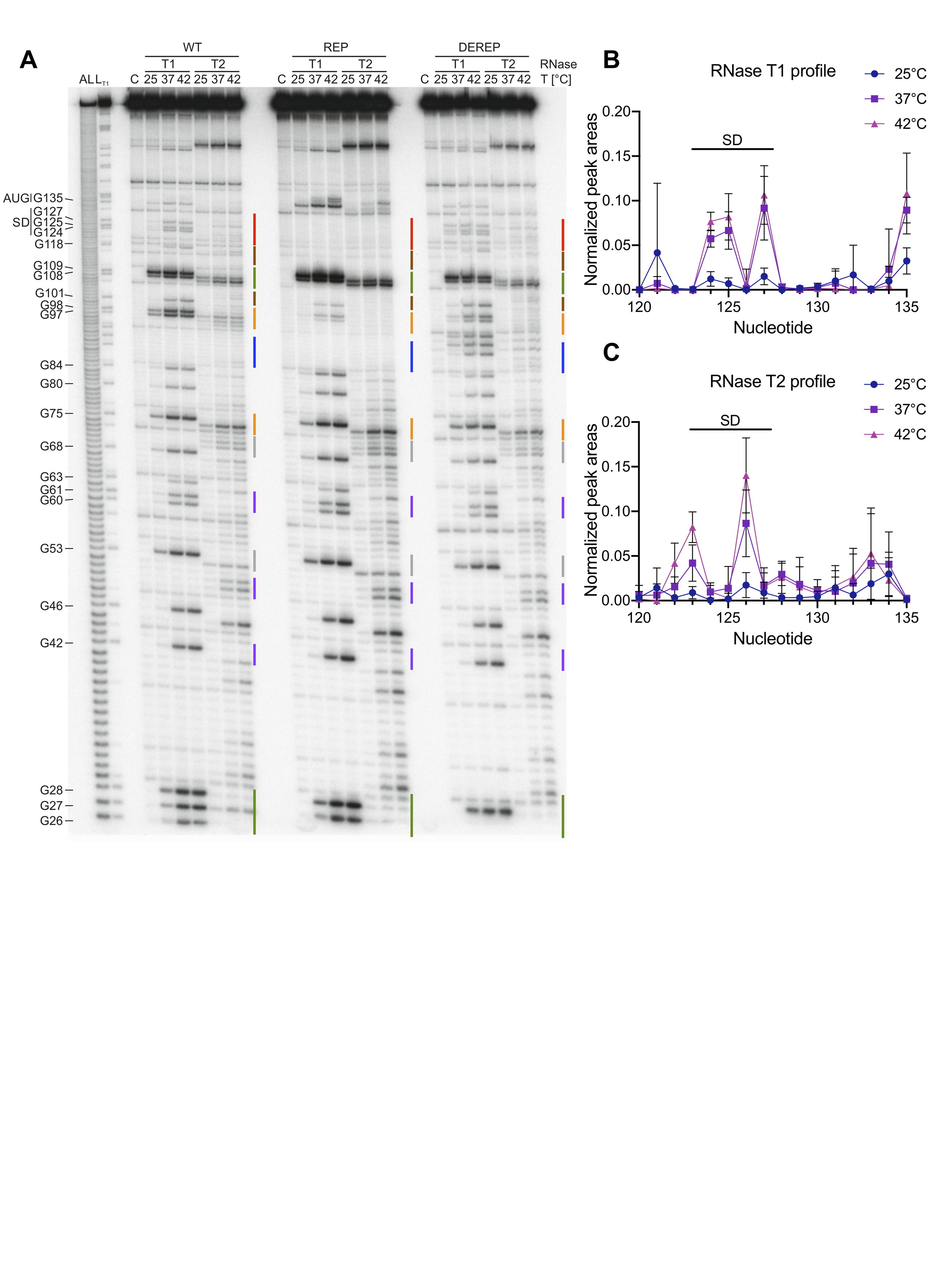

Supplement: S4 Fig — A) Entire gel from enzymatic structure probing shown in Fig 3A. 5’ end-labeled in vitro transcribed RNA containing the wildtype tviA 5’ UTR (WT), the rep3 (T90,92C) tviA 5’ UTR mutant (REP), or the derep4 (T89,91G;C93G) tviA 5’ UTR mutant (DEREP) was enzymatically probed with RNases T1 (cuts 3’ of single-stranded guanines) and T2 (cuts 3’ of single-stranded nucleotides with preference order: A > C > U > G) at 25, 37, and 42°C. Fragmented RNA was separated on an 8% polyacrylamide gel. AL: alkaline ladder. LT1: RNase T1 cleavage in sequence buffer at 37°C. C: RNA treated with water instead of RNase at 42°C. For many residues, the cleavage pattern fit the predicted secondary structure, with nucleotides susceptible to RNase T1 and T2 cleavage at 25°C (see also Fig 3B). These nucleotides are frequently located within predicted hairpin loops (e.g., GGG26-28, UAGG106-109; green highlight) or associated with inter-loop regions (e.g., GA74-75, GG97-98; orange highlight). The conformation of hairpin IB (see Fig 3B) deviates from the predicted structure because paired nucleotide stretches UG52-53 and GAAU68-71 were readily digested by the RNases at 25°C (gray highlight). Conversely, nucleotides UG41-42, AA49-50, and GG60-61, which were predicted to be unpaired, more likely adopt a double-stranded conformation because of increased RNase-mediated cleavage at 37°C and 42°C (purple highlight). B) Quantification of band intensities of RNase T1/T2 cleavage products in the region of the Shine-Dalgarno sequence of the wildtype tviA 5’ UTR at 25, 37, and 42°C. Data shown are representative of 4 independent experiments. (TIF) [file ppat.1009345.s009.tif]

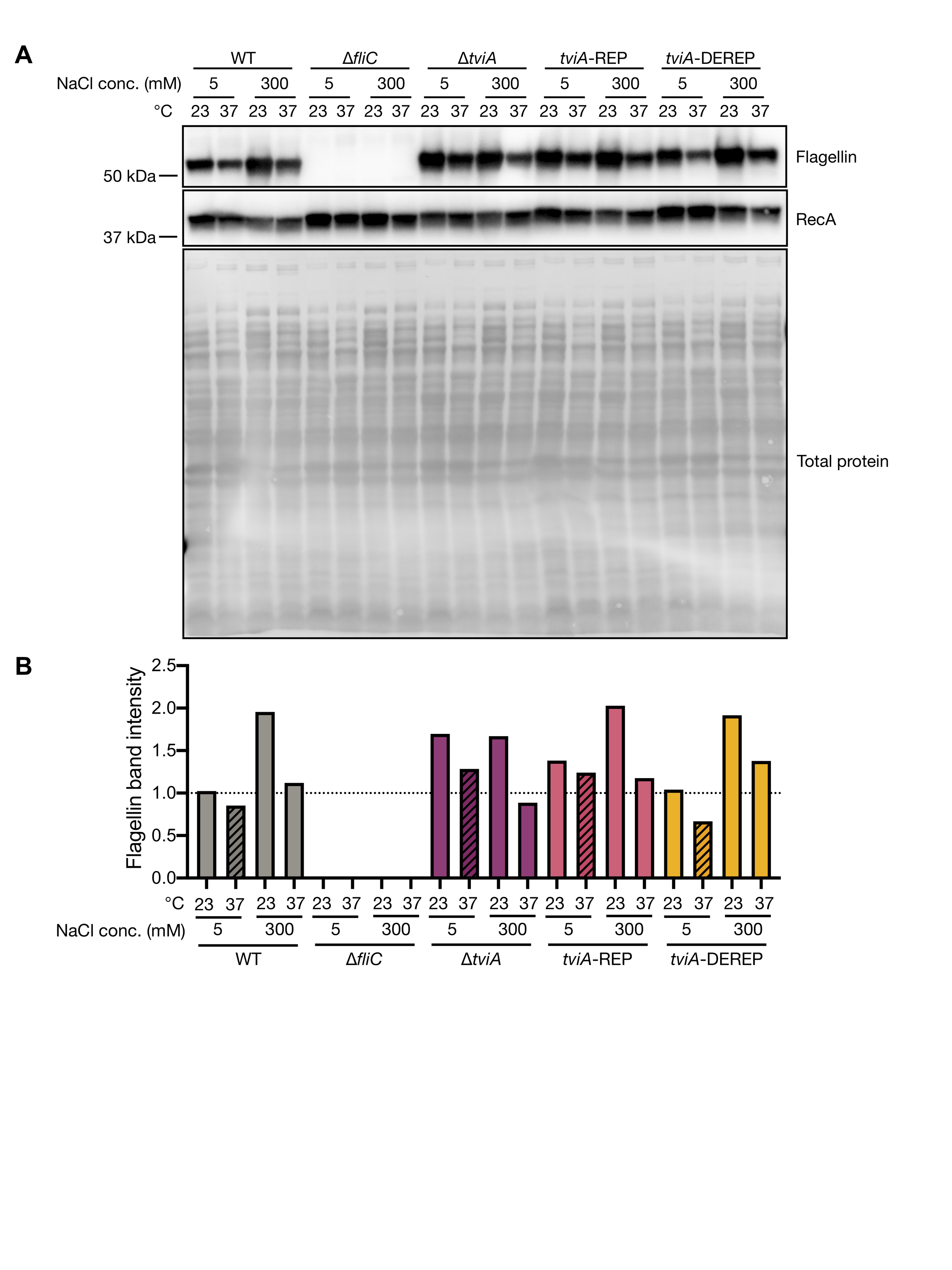

Supplement: S5 Fig — A) Entire Western blot with loading controls shown in Fig 5E. Whole cell lysates of WT, ΔfliC, ΔtviA, tviA-REP, or tviA-DEREP S. Typhi were probed for total protein, flagellin expression, and RecA expression after 24 hours of static growth under the following conditions: low salt media (5 mM NaCl LB broth) at 23°C, low salt media at 37°C, high salt media (300 mM NaCl LB broth) at 23°C, or high salt media at 37°C. B) Quantitation of flagellin expression from Western blot in S5A Fig. Flagellin band intensity was normalized to the RecA loading control band intensity for each sample to demonstrate similar results as seen with normalization to total protein (see Fig 5F). Data shown are representative of 2 independent experiments. (TIF) [file ppat.1009345.s010.tif]

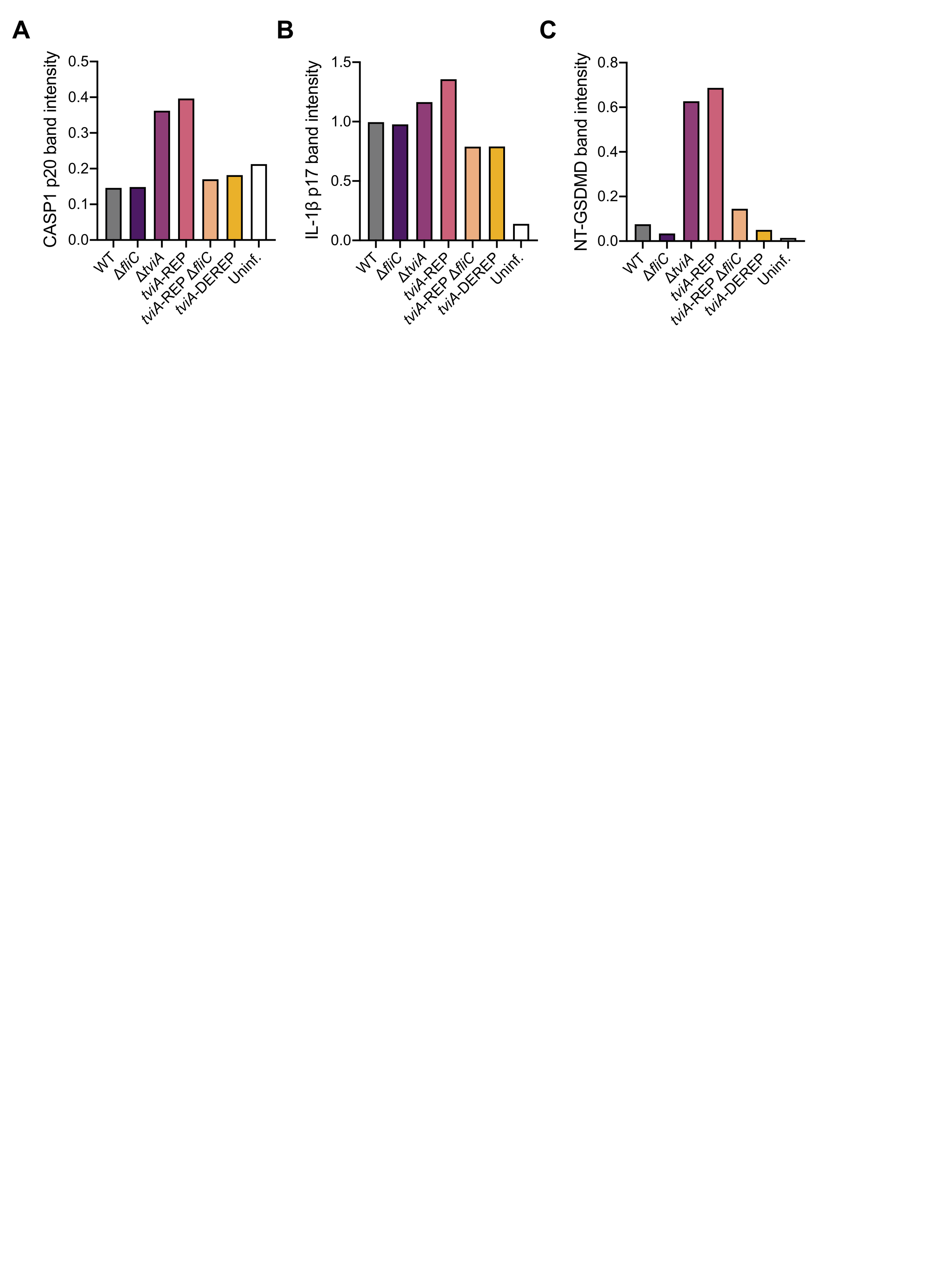

Supplement: S6 Fig — Quantitation of cleaved caspase-1 (A), cleaved IL-1β (B), and NT-GSDMD (C) from the Western blot shown in Fig 7I. Band intensities of each cleavage product were normalized to the band intensity of β-actin for each sample before being plotted. Data shown are representative of 2 independent experiments. (TIF) [file ppat.1009345.s011.tif]

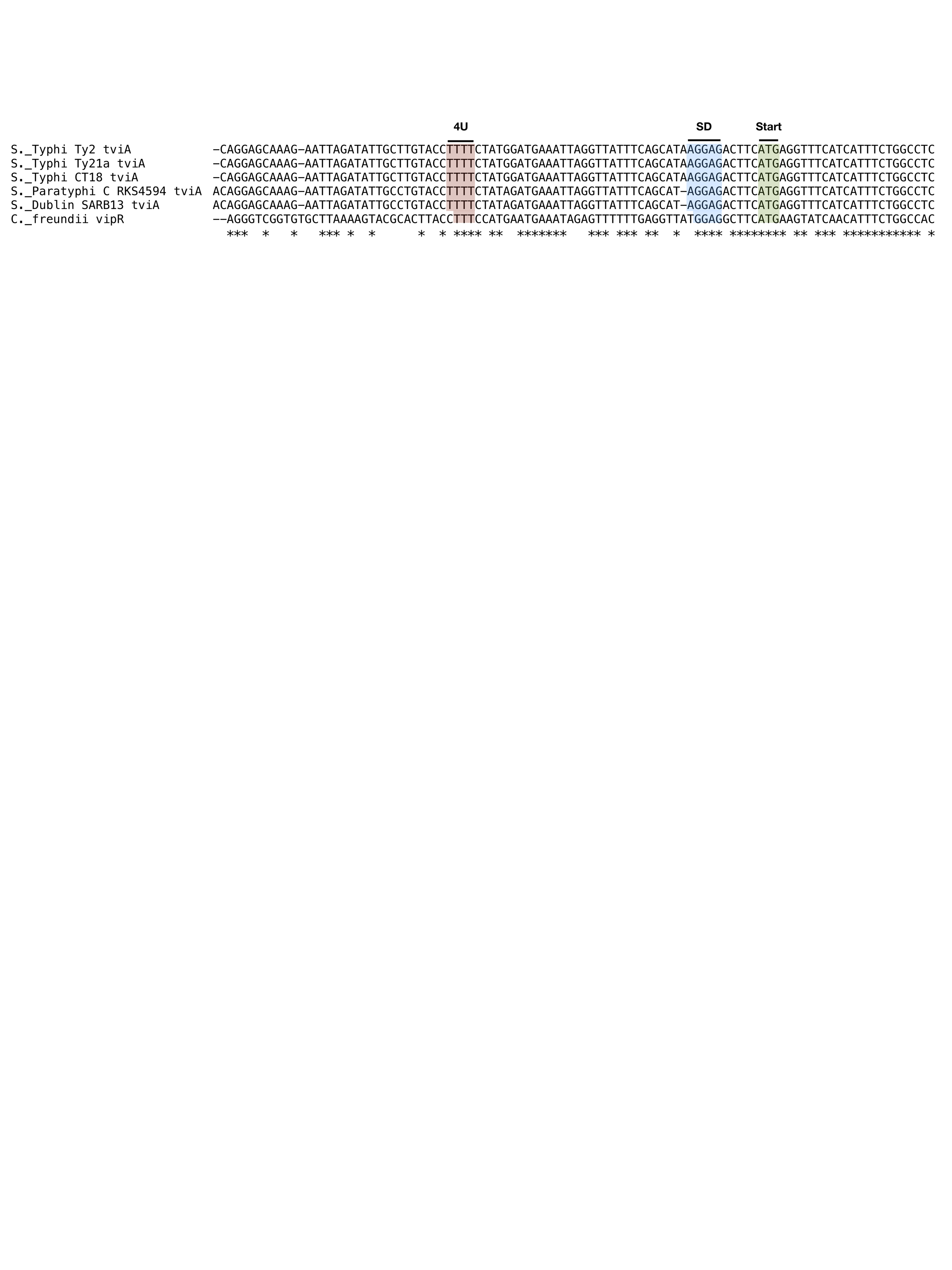

Supplement: S7 Fig — Alignment of the tviA (or equivalent homolog vipR in Citrobacter freundii) 5’ UTR nucleotide sequences from lab strain S. Typhi Ty2, vaccine strain S. Typhi Ty21a, multi-drug resistant clinical isolate S. Typhi CT18, clinical isolate S. Paratyphi C RKS4594, S. Dublin isolate SARB13, or opportunistic pathogen C. freundii reveals conservation of the fourU RNA thermosensor in enteric fever-causing human pathogens. FourU RNA thermosensor (4U) highlighted in red. Shine-Dalgarno (SD) highlighted in blue. Start codon highlighted in green. Asterisks indicate conserved residues between strains. (TIF) [file ppat.1009345.s012.tif]
