# Supplementary material for: A Salmonella Typhi RNA thermosensor regulates virulence factors and innate immune evasion in response to host temperature
Source: PLoS Pathog. 2021 Mar 2;17(3):e1009345. doi: 10.1371/journal.ppat.1009345 (PMC7954313; doi:10.1371/journal.ppat.1009345)
Supplement: Attachment — Submitted filename: Brewer_et al_2021_Response to reviewer comments.docx [file ppat.1009345.s013.docx]

Below is a point-by-point account of revisions made to address reviewers’ comments for the manuscript PPATHOGENS-D-20-02255 titled “A *Salmonella* Typhi RNA thermosensor regulates virulence factors and innate immune evasion in response to host temperature.” Our responses are in italics to distinguish them from reviewer comments.

Reviewer Comments (if any, and for reference):

Reviewer's Responses to Questions

**Part I - Summary**
Please use this section to discuss strengths/weaknesses of study, novelty/significance, general execution and scholarship.

Reviewer #1: In their study, Brewer et al. identify an RNA thermometer (RNAT) in the 5’UTR of the tviA mRNA of Salmonella Typhi, encoding a transcriptional master regulator of a well-defined regulon that comprises motility, virulence and immune evasion factors. Using a combination of genetics, RNA biochemical and phenotypic assays, the authors present mechanistic data that demonstrates that the tviA RNAT regulates translation initiation of TviA in a temperature-dependent manner, which in turn affects motility and Vi capsule expression of S. Typhi, and consequently, the host immune response to infection. Integrated with the previously known transcriptional induction of tviA through the OmpR/EnvZ two-component system, these new data suggest a compelling working model in which TviA expression is coupled to both, elevated temperature and low osmolarity, to ensure that the transition from an invasive, motile lifestyle to immune evasion occurs at the appropriate stage of the S. Typhi infection cycle.

The presented experiments were well-conducted and support the drawn conclusions. Further, the study is both, timely and relevant. I only have a few minor comments that I hope will be useful for the authors to further improve this interesting study.

Reviewer #2: Brewer et al describe the discovery and characterisation of an RNA thermosensor in the 5’ UTR region of the tviA gene of Salmonella Typhi. From the initial observation that the Vi polysaccharide expression was temperature sensitive but not as a result of transcriptional changes to the master regulator tviA, they discover a sequence with structural characteristics consistent with that of a RNAT. Through site directed mutagenesis they construct recombinant strains with altered structural properties that convincingly demonstrate that this is indeed an RNAT. The role of the RNAT in regulating expression of the viaB locus that affected the innate immune response and motility is then addressed.
This is a well written manuscript describing an excellent study that identifies a previously unknown regulatory level in the expression of an important virulence factor in S. Typhi. It is therefore highly novel and important piece of work. The only weakness is the use of three replicates throughout results in low statistical power. In general, this does not affect the major conclusions due to very low variance and large differences in the mean in most data. However, where means are similar, this may have resulted in falsely accepting the null hypothesis.

*We have addressed this concern below.*

Reviewer #3: (No Response)

**Part II – Major Issues: Key Experiments Required for Acceptance**
Please use this section to detail the key new experiments or modifications of existing experiments that should be absolutely required to validate study conclusions.

Generally, there should be no more than 3 such required experiments or major modifications for a "Major Revision" recommendation. If more than 3 experiments are necessary to validate the study conclusions, then you are encouraged to recommend "Reject".

Reviewer #1: (No Response)

Reviewer #2: (No Response)

Reviewer #3: Manuscript by Brewer et al investigates the role of 5’ UTR region of tviA, a transcriptional regulator of the critical virulence factors Vi  capsule, flagellin, and type III secretion system-1 expression, of S. Typhi. Authors identify a RNA thermosensor (RNAT) in the UTR region and show that this region post-transcriptionally controls the TviA protein. Using in vitro reporter system in E. coli and point mutations in the tviA 5’ UTR that either stabilize or destabilize the RNAT structure, the authors RNAT in UTR region is closed therefore the tviA is repressed at low temperatures. Authors also show that the RNAT melts open at higher temperatures allowing ribosome access and the translation of tviA. Authors go on to show that this system controls the expression of Vi and repression of flagella in a temperature dependent way. However, the growth in high salt (150mM NaCl) conditions can override and induce maximal suppression of flagellin expression.By utilizing mutants where the RNAT structure is stabilized or destabilized, authors show that the regulation of flagellin expression would be detected by the host inflammasome in THP-1 macrophages. Overall this is an elegant investigation of the temperature dependent regulation of tviA. The manuscript is well written. My only concern is the big conclusion drawn in Fig 8 is not fully supported. Although the RNAT function was investigated in high and low temperature or in high and low osmolarity, these experiments were done separately. To conclude the combinatorial effect of these conditions, authors should perform the experiments in the four conditions as proposed in Fig 8, high osmolarity and low temperature, high osmolarity and high temperature, low osmolarity and low temperature, low osmolarity and high temperature. There are no in vivo experiments to support the hypothesis in Fig 8 but at least if the in vitro experiments can mimic what might be happening in vivo, this would increase the impact of the findings

*We thank Reviewer 3 for this suggestion and now include data to further support the conclusion drawn in Figure 8 (please see Fig. 5E and F and Fig. S5). We grew the WT, ΔfliC, ΔtviA, tviA-REP, and tviA-DEREP S. Typhi strains statically under the following conditions: low salt (5 mM NaCl LB broth) at 23°C, low salt at 37°C, high salt (300 mM NaCl LB broth) at 23°C, and high salt at 37°C. We then assessed flagellin production via Western blot. As expected, WT S. Typhi had the lowest levels of flagellin under low salt high temperature conditions and the highest levels of flagellin under high salt low temperature conditions. The remaining two tested growth conditions produced intermediate flagellin levels. These results support our conclusion that regulation of TviA production is a two-checkpoint system, where osmolarity controls tviA transcription and temperature controls translation. As posited in Figure 8, only under conditions of low osmolarity and high temperature should flagellin be maximally suppressed. The results we present in Figure 5E and F and Figure S5 demonstrate the greatest suppression of flagellin under low salt high temperature conditions and support the model we describe in Figure 8. These in vitro conditions mimic the conditions S. Typhi experiences once it has disseminated systemically in a new host during acute infection, and the maximal suppression of flagellin under these conditions enables S. Typhi to evade innate immune detection. Given that S. Typhi is a human-restricted pathogen, it is difficult to assess S. Typhi pathogenesis with in vivo experiments, especially because the viaB locus, and tviA specifically, are not present in the highly related pathogen S. Typhimurium, which does successfully infect mice and manifest as a typhoid fever-like disease. However, we believe that the in vitro data we present in Figures 5E and F and S5 bolster our conclusions described in Figure 8, and the inclusion of the RNAT mutant strains in the experiments presented in Figure 5E,F and S5 further illustrates the importance of the tviA RNAT in modulation of flagellin expression.*

*For newly included data and descriptions, please see lines 405-412, 436-477, and 1469-1478.*

**Part III – Minor Issues: Editorial and Data Presentation Modifications**
Please use this section for editorial suggestions as well as relatively minor modifications of existing data that would enhance clarity.

Reviewer #1: • Experimental data were typically collected over several independent experiments, each performed in technical replicates. However, it seems as if for the presentation of the data often times just one (representative) biological replicate with all its technical replicates was shown (e.g. Fig. 1). Unless there is a reason against it (e.g. large donor variability for the assays with primary human macrophages), I would however prefer plotting individual biological – rather than technical – replicates.

*Reviewer 1 has correctly pointed out that it is not possible to combine the results of the primary human macrophage experiments due to large variability between responses of individual donors. And although our other experiments utilize more controlled systems than primary cells, certain assays demonstrate large variability between repeats, precluding us from combining results across repeats. However, the phenotypes that we observed between the different tested conditions within each assay are consistent across repeats. For example, for Figure 1C, we consistently see a significant increase in Vi capsule expression in WT S. Typhi following a temperature shift to 37°C for 30 minutes across three independent experiments (see below). We can combine all these individual datapoints from the three independent experiments and still see the significant difference in this phenotype, despite variability between each repeat (see below; symbol shapes correspond with independent repeats). However, if we take the mean of the replicates from each independent repeat and only plot those, the phenotype no longer appears significant due to the high variability between assay repeats (see below). This does not mean that the phenotype we observed is not significant. Rather it means that the assay has variability between repeats.*

*Where possible due to low variability between assay repeats, we have updated the figures to include all the datapoints from all repeats for the sake of data transparency. Updated figures include: Figure 1C and D, Figure 2E, Figure 5B.*

• Fig. 2E: Do the authors have an explanation for the (RNAT-independent) increase of bgaB mRNA levels at 42°C? Unless the pBAD promoter responds to temperature, this would imply that the mRNA is more stable in the warmth.

*We hypothesize two potential explanations for this observation: 1) the promoter may be somewhat temperature-sensitive in E. coli, or 2) the high temperature of 42°C may increase E. coli membrane fluidity, allowing for greater uptake of arabinose at this temperature, and, thus, greater transcriptional induction. Although one could argue that this suggests the increased β-galactosidase activity observed in the WT and derepressed mutant constructs at 42°C is due to increased bgaB transcript levels, we also see significantly higher levels of bgaB mRNA transcript at 42°C with the repressed mutant construct, yet no correspondingly high β-galactosidase activity. Because we observed increased mRNA levels at 42°C for all the tested tviA RNAT variants, this suggests that the differences we observed in β-galactosidase activity are due to the mutations in the 5’ UTRs of these variants affecting translation rather than mRNA transcript levels. Please note, we have also updated Figure 2E to include all the datapoints from the two independent experiments, which reduces the significance of some of the phenotypes but still demonstrates the same increased trend at 42°C.*

• Fig. 7A, D, E: Some data points are below the 0% line. How can the percentage of cell death be negative?

*This has to do with how the % cell death is calculated to account for background signal from the lactate dehydrogenase release assay. In order to calculate a “% cell death,” the field frequently uses the following formula to normalize absorbance readings:*

$$\frac{Experimental absorbance-Untreated absorbance average}{Lysis absorbance average-Untreated absorbance average}*100$$

*If some of the raw experimental absorbance values fall below the average of the untreated (or in this case “Uninfected”) values because there is minimal cell death in that sample, this can return a negative % cell death value. Please note that this formula is described in the Materials and Methods section (see lines 1091-1094).*

• Western blot in Fig. 7I: The effect of mutating the tviA RNAT (‘tvi-REP’) or deleting the entire gene (‘ΔtviA’) is less clear on the CASP1 and IL1b band signals than it is on GSDMD. Could the authors quantify band intensities for this western blot (as they do for the toeprinting assay in Fig. 4B, C)?

*Thank you for this suggestion. We have now quantitated the band intensities for the cleavage products in Figure 7I and they are presented in Figure S6. As Reviewer 1 suggested, this helps clarify the phenotypic differences observed in inflammasome activation upon infection with these different S. Typhi strains.*

*Additionally, we realized that the nonspecific band observed in the NT-GSDMD blot in the initial manuscript submission is caused by the pro-IL-1β band from previous probing, despite stripping the blot between probing with different antibodies. To greater distinguish the NT-GSDMD band, we re-ran the same samples on a new gel, blotted, and probed only with the anti-NT-GSDMD antibody followed by β-actin. Figure 7I has been updated with the new NT-GSDMD and corresponding β-actin data and more clearly demonstrates the higher levels of NT-GSDMD observed following infection with the ΔtviA or tviA-REP S. Typhi mutants.*

Text edits:
• Line 159: In the main text, the authors use a different numbering system (“position 123 to 127 within the 5’UTR”) than in the referenced figure (“-132 nt to +3 nt from AUG”; line 170 and Fig. 2A). For reasons of consistency, this may be homogenized.

*Thank you for catching this inconsistency. We have updated the legend for Figure 2A to state “132 nt plus AUG codon; 135 nt total” so that the positions 123 to 127 mentioned in the text are consistent with the figure legend. Please see lines 193-194.*

• Lines 184 and 375: Please correct “16S mRNA” to “16S rRNA”.

*Thank you for catching this. The text has been corrected.*

• Lines 279, 281, 295, 296: The authors may want to label stems/hairpins I and II in Fig. 2A to allow the readers to better follow these text parts.

*Figure 3B is the same predicted secondary structure as shown in Figure 2A, but it is annotated with the stem/hairpin labels and cleavage information based on the structure probing gel. In order to assist the reader in following the text and referring to the predicted secondary structure, we have included more references in the text to refer back to Figure 3B and 3C, where appropriate. Please see lines 303, 306, 319.*

• Lines 339-341: Please specify to which UTR variant the quantifications in B and C refer. (I assume it’s the wild-type, but since there are additional UTR variants depicted on the gel in panel A, it should probably be mentioned explicitly.)

*Thank you for catching this omission. We are referring to WT in these lines, and we have updated the figure legend to explicitly state that. Please see lines 363-364.*

• The names of the bacterial mutant strains in Fig. 7D and E should be italicized (as done in the remaining panels of this figure).

*Thank you for catching this. We have updated the figures to have proper italicization.*

Reviewer #2: General comment
1. Data in figure 1 includes treatment at 23C and later experiments equivalent low temperature treatments are at 25C. Is this a typo, or were the experiments really performed at different temperatures and if so was there a rationale for this change?

*Low temperature culture conditions were performed at ambient temperature. Depending on the location where the experiment was performed, the average ambient temperature ranged from 23°C to 25°C. We have updated the Materials and Methods section to more explicitly state this (please see lines 814-816).*

2. At what temperature does the RNAT de-repress translation of TviA?

*To more precisely answer this question, we would need to perform additional experiments (e.g., β-galactosidase activity assays) with more temperatures at smaller increments. Our data suggests that there is a gradual unzipping, or opening, of the RNAT with increasing temperature. Thus, it might be hard to pinpoint an exact temperature where translation of TviA transitions between off and on because this process rather operates on a gradient. Even at the low temperatures tested, we observed low levels of Vi capsule expression (Fig. 1C and 5A), β-galactosidase activity (Fig. 2C and D), RNase cleavage (Fig. 3A and S4), and ribosome binding (Fig. 4A) from the WT 5’ UTR of tviA. These data suggest that there is a low level of translation occurring even at the lowest tested temperature. Thus, even colder temperatures may further zip up the RNAT, leading to even less translation.*

Specific comments

3. 156 'S' should be italics.

*We have corrected this typo.*

4. 156-158. Please describe the use of Mfold in Materials and Methods.

*We have included a section in the Materials and Methods describing the use of Mfold. Please see lines 818-820.*

5. 162-167. Please make it clear why these structural elements were indicative of a cis-regulatory thermosensor. Suggest citing studies in which similar structures were shown to have these functions.

*Thank you for this suggestion. We have now included a description in the text and citations to make it more clear as to why these structural elements are indicative of a cis-regulatory thermosensor. Please see lines 184-188.*

6. 588-590. These are new data analysis and I suggest that they are included in the results section. The tviA 5' UTR of S. Paratyphi C and S. Dublin are included in the alignment in Figure S5 and should also be mentioned in the text. Currently only the presence of the genes in these serovars is mentioned.

*Thank you for this suggestion. We have moved the alignment and accompanying description to the results section of the manuscript. Please see lines 605-630.*

7. 163. Please comment on whether the presence of an additional adenine in the S. Typhi sequence (bulging adenine) that is absent in Dublin and Paratyphi C would be expected to affect the thermostability of the secondary structure. Would you expect this to affect thermoregulation in Paratyphi C and Dublin?

*We hypothesize that the lack of the bulging adenine immediately 5’ to the SD in S. Dublin and S. Paratyphi C tviA 5’ UTR sequences would stabilize the RNAT structure, meaning that it would require a higher temperature for the structure to melt open and enable translation. However, we cannot definitively conclude the effects of the bulging adenine on the thermoregulatory properties of the tviA 5’ UTR RNAT without experimental testing.*

*We have included a comment on the potential effects of the bulging adenine in the manuscript. Please see lines 621-627.*

8. 809. Please provide information on the source of 30S ribosomal subunit

*Details about the source of the 30S ribosomal subunit have been added to the Materials and Methods section. Please see lines 895-896.*

9. 956-959. A description of how relative expression levels were calculated should be included. Presumably this was the delta delta Ct method?

*Reviewer 2 is correct that we used the ΔΔCt method to calculate relative expression levels. A description of the use of this method has been included in the Materials and Methods section. Please see lines 1038-1041.*

Reviewer #3: No minor issues detected.
